# HNRNP A1 Promotes Lung Cancer Cell Proliferation by Modulating *VRK1* Translation

**DOI:** 10.3390/ijms22115506

**Published:** 2021-05-23

**Authors:** Hye Guk Ryu, Youngseob Jung, Namgyu Lee, Ji-Young Seo, Sung Wook Kim, Kyung-Ha Lee, Do-Yeon Kim, Kyong-Tai Kim

**Affiliations:** 1 Department of Life Sciences, Pohang University of Science and Technology (POSTECH), Pohang 37673, Korea; hyegukryu@gmail.com; 2 Division of Integrative Biosciences and Biotechnology, Pohang University of Science and Technology (POSTECH), Pohang 37673, Korea; ysjung@postech.ac.kr (Y.J.); jyseo@postech.ac.kr (J.-Y.S.); kimsw@postech.ac.kr (S.W.K.); 3 Department of Molecular, Cell and Cancer Biology, University of Massachusetts Medical School, Worcester, MA 01065, USA; Namgyu.Lee@umassmed.edu; 4 Division of Cosmetic Science and Technology, Daegu Haany University, Gyeongsan 38610, Korea; kyungha.lee@dhu.ac.kr; 5 Department of Pharmacology, School of Dentistry, Kyungpook National University, Daegu 41940, Korea; dykim82@knu.ac.kr

**Keywords:** HNRNP A1, VRK1, lung cancer, post-transcriptional regulation, 3′UTR

## Abstract

THeterogeneous nuclear ribonucleoprotein (HNRNP) A1 is the most abundant and ubiquitously expressed member of the HNRNP protein family. In recent years, it has become more evident that HNRNP A1 contributes to the development of neurodegenerative diseases. However, little is known about the underlying role of HNRNP A1 in cancer development. Here, we report that HNRNP A1 expression is significantly increased in lung cancer tissues and is negatively correlated with the overall survival of patients with lung cancer. Additionally, HNRNP A1 positively regulates vaccinia-related kinase 1 (VRK1) translation via binding directly to the 3′ untranslated region (UTR) of *VRK1* mRNA, thus increasing cyclin D1 (CCND1) expression by VRK1-mediated phosphorylation of the cAMP response element–binding protein (CREB). Furthermore, HNRNP A1 binding to the *cis*-acting region of the 3′UTR of *VRK1* mRNA contributes to increased lung cancer cell proliferation. Thus, our study unveils a novel role of HNRNP A1 in lung carcinogenesis via post-transcriptional regulation of VRK1 expression and suggests its potential as a therapeutic target for patients with lung cancer.

## 1. Introduction

Lung cancer is one of the most common types of cancer and the overall cure and survival rates for patients with lung cancer remain low [1,2,3]. Targeted agents against different components of cell cycle-related protein kinases, such as cyclin-dependent kinase, polo-like kinase, checkpoint kinase and aurora kinase, have become attractive targets for anti-cancer therapy because tumor cell proliferation is frequently associated with genetic or epigenetic alterations in key regulators of the cell cycle [4]. However, in clinical trials, due to side effects and limited responses, the preclinical promise of these drugs for patients with lung cancer was not realized [5]. Thus, identification of novel cell cycle-specific target proteins and development of their selective inhibitors are required.

Vaccinia-related kinase 1 (VRK1), a mitotic serine/threonine kinase, plays an important role in cell cycle progression by participating in wide variety of cell division processes. In cancer progression, VRK1 promotes the G1/S transition through phosphorylating the cAMP response element (CRE)-binding protein (CREB), thereby enhancing the binding affinity of CREB to the cyclin D1 (*CCND1*) promoter [6]. Additionally, VRK1 phosphorylates barrier-to-autointegration factor (BAF) in early mitosis and is involved in nuclear envelope (NE) dynamics, such as assembly and disassembly. In the mitotic phase, VRK1 affects histone modification by phosphorylating histone H3. Furthermore, VRK1 is a well-known DNA damage repair protein that phosphorylates 53BP1 [7], NBS1 [8] and histone H2AX [9], suggesting that VRK1 confers resistance to DNA damage by affecting the DNA damage response. Consequently, these critical roles suggest that VRK1 could be an excellent candidate for cancer therapy and recent works have focused on VRK1 as a possible drug target for cancer treatment [10,11]. VRK1 is overexpressed in several types of cancers, including non-small cell lung cancer; however, there is a lack of data on the control of *VRK1* gene expression, primarily regarding the mechanism of post-transcriptional control of VRK1 expression.

Heterogenous nuclear ribonuclearprotein (HNRNP) A1 is a member of the HNRNP family, which plays a major role in the biogenesis and expression of mRNA. HNRNP A1 plays key steps in mRNA metabolism such as alternative splicing, mRNA export, translation, microRNA processing and telomere maintenance. HNRNP A1 has structurally and functionally independent domains; two closely related RNA-binding domains (RBDs) of the RNA recognition motif (RRM) type (RRM1 and RRM2), collectively referred to as unwinding protein 1 (UP1) and a highly flexible RGG-rich C-terminal region. UP1 is the primary RNA-binding domain, whereas the C-terminus mediates homologous and heterogeneous protein–protein interactions. Emerging evidence has revealed the essential function of HNRNP A1 in brain function. A previous study indicated that HNRNP A1 has a role in Alzheimer disease because it is involved in the maturation of amyloid precursor protein (*APP)* mRNA [12]. Additionally, HNRNP A1 may be associated with a variety of pathogeneses and symptoms of spinal muscular atrophy (SMA) [13,14] and amyotrophic lateral sclerosis (ALS). Nuclear HNRNP A1 immunoreactivity was lost or weak in the nucleus of spinal motor neurons of patients with ALS [15]. Recently, several studies have indicated the overexpression of HNRNP A1 in several tumor types, including lung [16], breast [17] and colon cancer [18]. However, the functional relevance of HNRNP A1 in cancer and tumorigenesis still remains unclear.

In this study, we investigated the regulation of *VRK1* gene expression and assessed the role of HNRNP A1 in the proliferation of the lung cancer cell. We found upregulation of the RNA-binding protein (RBP) HNRNP A1 in human lung cancer. The overexpression of HNRNP A1 was significantly associated with lung cancer progression and poor survival features. Mechanistically, we identified the oncogene *VRK1* as a critical target of HNRNP A1. HNRNP A1 upregulated *VRK1* translation via binding to its 3′ untranslated region (UTR). In addition, we applied CRISPR/CAS9 systems to understand *cis*- and *trans*-regulatory elements in HNRNP A1/VRK1 signaling pathway, which may represent therapeutic targets for human lung carcinogenesis. In short, our data demonstrated that HNRNP A1 is an important pro-tumorigenic factor in lung carcinogenesis and the therapeutic targeting of HNRNP A1/VRK1 may offer options for human lung cancer intervention.

## 2. Results

### 2.1. VRK1 3′UTR Is Involved in Translation

**Figure 1:**
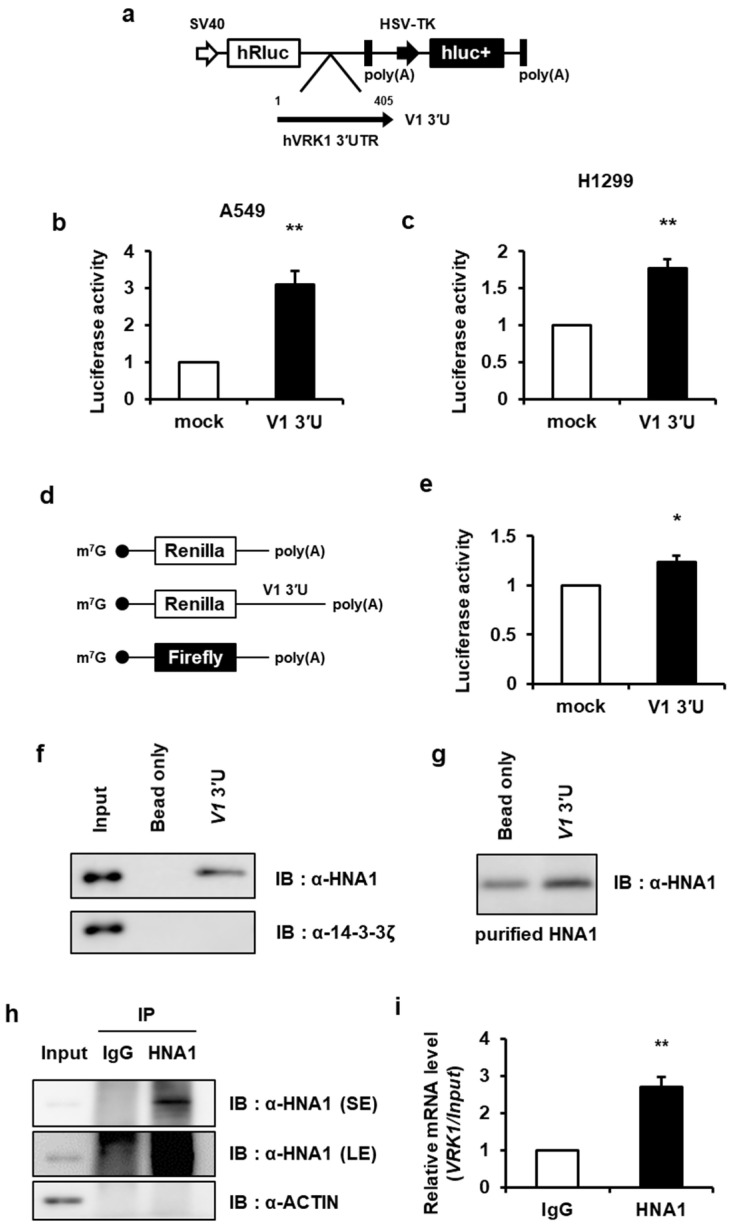
The 3′UTR of *VRK1* is involved in translation and HNRNP A1 directly interacts with the 3′UTR of *VRK1* mRNA. (**a**) Schematic drawing of reporter gene construct is shown. Reporter construct is composed of the psiCHECK2-backbone plasmid that encodes the Renilla luciferase gene followed by the human 3′UTR of *VRK1* (V1 3′U). (**b**,**c**) Levels of reporter activities are measured by analysis of *VRK1* 3′UTR (V1 3′U) luciferase reporter. Luciferase activity is shown as the ratio of hRluc to hluc+ and the luciferase activity of mock was set as 1. Bars represent means ± s.e.m. (unpaired two-tailed Student’s *t*-test, *n* = 4; ** *p* < 0.01). (**d**) Schematic representation of the mRNAs measured in this experiment are shown. Firefly mRNA reporters were also used for normalization. (**e**) Mock and *VRK1* 3′U mRNAs were synthesized in vitro by T7 RNA polymerase and were transfected into A549 cells using Lipofectamine 2000. After a 12-h incubation, luciferase assays were performed (unpaired two-tailed Student’s *t*-test, *n* = 4; n.s., not significant; * *p* < 0.05). (**f**) The in vitro-transcribed *VRK1* 3′UTR construct was labelled with biotin-UTP and was incubated with cell lysates of HEK293A cells. Biotin-UTP labelled *VRK1* 3′UTR was pulled down with streptavidin bead. Then, streptavidin affinity purified samples were separated by SDS-PAGE and were subjected to immunoblot analysis with the indicated antibodies. (**g**) To prove the direct interaction of HNRNP A1 and *VRK1* 3′UTR, biotin-conjugated *VRK1* 3′UTR was prepared by in vitro transcription and was incubated with non-tagged purified HNRNP A1. Then, the biotin-conjugated *VRK1* 3′UTR was pulled down using streptavidin, which were later subjected to immunoblot analysis with the indicated antibodies. (**h**) Western blot analysis for HNRNP A1 and ACTIN in HEK293A cell lysate after IP with mouse anti-HNRNP A1 or mouse IgG shows enrichment for HNRNP A1 and depletion of other proteins, as indicated by absence of ACTIN in IP fractions. (**i**) Endogenous HNRNP A1 binds to endogenous *VRK1* mRNA. Lysates from HEK293A cells were used for RNA immunoprecipitation (RNA-IP) analysis using IgG and anti-HNRNP A1 antibodies. RNA abundance in IP samples was determined by qRT-PCR. Bars represent means ± s.e.m. (unpaired two-tailed Student’s *t*-test, *n* = 3; ** *p* < 0.01).

For the last decade, research to better understand the molecular pathways involved in the control of gene expression in cancer cells has largely focused on post-transcriptional regulation by RNA-binding proteins (RBPs). To investigate post-transcriptional regulation of VRK1 in lung cancer, our initial aim was to identify whether VRK1 expression in lung cancer cells utilizes 3′UTR-mediated translation because the UTRs act as targets for most post-transcriptional regulation. First, we determined whether the 3′UTR of *VRK1* harbors translation activity using a luciferase reporter system. The psiCHECK2 vector contains two luciferase genes, renilla (hRluc) as the experimental reporter and the control firefly reporter (hluc+) as a transfection control. This vector is designed to monitor changes in the expression of a target gene fused to a reporter gene. *VRK1* 3′UTR was inserted between the two reporter genes, hRluc and hluc+ and the ratio of hRluc/hluc+ was measured in A549 and H1299 lung cancer cell lines as a representation of translation activity (Figure 1a). *VRK1* 3′UTR showed profound translation activity compared with those of the controls (Figure 1b,c). To exclude any transcriptional effect of *VRK1* 3′UTR and further assess the potential translation enhancing activity of *VRK1* 3′UTR, we transfected A549 with mRNA of a reporter construct with an m^7^GpppG-cap followed by the luciferase gene and *VRK1* 3′UTR (Figure 1d). We observed that luciferase synthesis from the *VRK1* 3′UTR-containing reporter was considerably higher than the synthesis from the mock reporter (Figure 1e). Together, these results demonstrate that *VRK1* 3′UTR indeed possesses translation stimulation activity.

### 2.2. HNRNP A1 Interacts with VRK1 mRNA and Enhances Its translation

The *VRK1* mRNA and protein levels increase gradually from the G1 phase to the early mitotic phase [19]. Additionally, VRK1 plays an essential role in cancer progression by controlling the cell cycle regulators associated with G1/S transition [20]. In line with these important roles of VRK1 during G1/S in cancer cell progression, accumulating evidence has suggested that HNRNP A1 is another critical regulator of cell cycle progression. For example, it was reported that the suppression of HNRNP A1 inhibits lung adenocarcinoma cell proliferation through cell cycle arrest at the G0/G1 phase [21]. In addition, VRK1 and HNRNP A1 are frequently overexpressed in several human cancers, including hepatocellular carcinoma (HCC) and the overexpression of VRK1 and HNRNP A1 is correlated with a poor prognosis for HCC patients [17]. Arginine methylation of the hnRNP A1 and a family of protein arginine methyltransferases (PRMTs) are found to be overexpressed in breast, prostate and colon cancer cells [22]. One study showed an increased expression level of HNRNP A1 in cervical cancer cells including HeLa, providing evidence that the expression of HNRNP A1 is closely related to HeLa cell proliferation, invasion and migration [23]. Therefore, we investigated the clinical significance of HNRNP A1 as one of the RNA-binding proteins in the regulation of *VRK1* mRNA.

To obtain more direct evidence for the regulatory role of HNRNP A1 on *VRK1* mRNA regulation, we verified an interaction between the *VRK1* 3′UTR and HNRNP A1 using streptavidin-biotin RNA affinity purification analysis. We found that HNRNP A1 was co-precipitated with biotin-labeled *VRK1* 3′UTR mRNA (Figure 1f). It is possible that the result of the in vitro binding assay using cell lysates was due to an indirect RNA-protein interaction. Thus, to determine whether HNRNP A1 binds directly to the *VRK1* 3′UTR, we performed the biotinylation assay and immunoblotting using in vitro purified HNRNP A1 protein. The non-tagged purified HNRNP A1 was bound to the biotin-labeled *VRK1* 3′UTR (Figure 1g). To probe whether HNRNP A1 binds to *VRK1* mRNA in vivo, we performed RNA-immunoprecipitation (RNA-IP) with a HNRNP A1 antibody, followed by quantitative RT-PCR (qRT-PCR). Consistent with our hypothesis, *VRK1* transcripts were found to be enriched in HNRNP A1-specific RNA-IPs (Figure 1h,i). These data identify HNRNP A1 as a strong candidate for the *trans*-acting factor that interacts with the *VRK1* 3′UTR.

**Figure 2:**
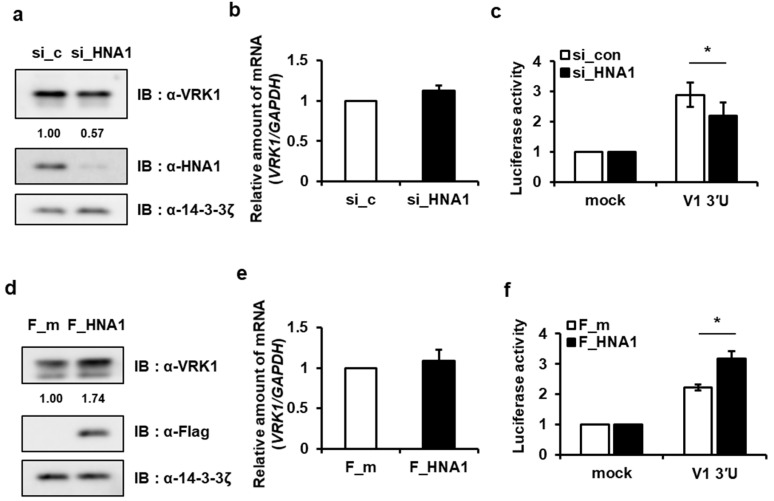
HNRNP A1 regulates the translation of *VRK1* via its 3′UTR. (**a**) Immunoblots for HNRNP A1 and VRK1 in A549 cells at 36–48 h post-transfection of either control siRNA (si_c) or siRNA targeting HNRNP A1 (si_HNA1) are shown. 14-3-3ζ was used as a loading control. VRK1 protein abundance is decreased in HNRNP A1 knocked-down cells. (**b**) Levels of the *VRK1* mRNA was measured by qRT-PCR. Values are means ± s.e.m. (unpaired two-tailed Student’s *t*-test, *n* = 3; n.s., not significant). (**c**) HNRNP A1 silencing inhibited *VRK1* 3′UTR luciferase activity in A549 cells (two-way ANOVA with Sidak’s multiple comparisons, *n* = 5; * *p* < 0.05). (**d**,**e**) After transfecting A549 cells with either Flag_mock (F_m) or Flag_HNRNP A1 (F_HNA1), the levels of VRK1, Flag and loading control, 14-3-3ζ, were analyzed by Western blot analysis (**d**) while the levels of *VRK1* mRNA were measured by qRT-PCR analysis (**e**). Values are means ± s.e.m. (unpaired two-tailed Student’s *t*-test, *n* = 3; n.s., not significant). (**f**) Enhancement of *VRK1* 3′UTR luciferase activity in HNRNP A1 overexpressing cells is shown as a bar graph (two-way ANOVA with Sidak’s multiple comparisons, *n* = 5; * *p* < 0.05).

We clearly confirmed that translation activity resides within the *VRK1* 3′UTR and that HNRNP A1 binds to this region. To investigate the possible role of HNRNP A1 as a *trans*-acting factor that enhances VRK1 translation, we transfected A549 cells with a siRNA that targets HNRNP A1 and confirmed that this results in a decreased level of VRK1 protein (Figure 2a). Then, we found that the level of *VRK1* mRNA was not significantly changed in HNRNP A1 knockdown cells (Figure 2b). To determine whether HNRNP A1 regulates *VRK1* 3′UTR-mediated translation, we measured *VRK1* 3′UTR reporter activity in HNRNP A1 knockdown cells. We found that *VRK1* 3′UTR reporter activation was decreased in cells with HNRNP A1 knockdown (Figure 2c). Additionally, we confirmed the function of HNRNP A1 using overexpression in A549 cells. Although the overexpression of HNRNP A1 did not alter the mRNA level of *VRK1,* it did increase endogenous VRK1 protein levels and the reporter activity of *VRK1* 3′UTR (Figure 2d–f).

The effect of HNRNP A1 on 3′UTR-mediated translation enhancement was once again confirmed in another lung cancer cell line, H1299, which were used to create HNRNP A1-depleted and HNRNP A1- overexpressing cells (Figure S1a–g). To exclude the possibility that HNRNP A1 was affecting *VRK1* mRNA stability, we treated cells with the transcription inhibitor actinomycin D (Act.D) and measured endogenous *VRK1* mRNA levels in the presence of si_HNRNP A1. After Act.D treatment, *VRK1* mRNA decay kinetics was unaffected by HNRNP A1 knockdown (Figure S1h). These results suggest that HNRNP A1 acts as a crucial *trans*-acting factor for 3′UTR-mediated *VRK1* mRNA translation.

### 2.3. A Stem-Loop Containing a HNRNP A1 Binding Site Is a Cis-Acting Element in VRK1 3′UTR-Mediated Translation

**Figure 3:**
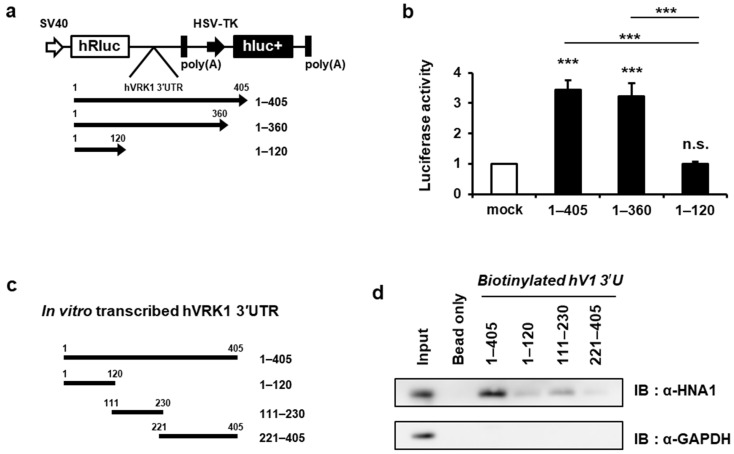
Secondary loop containing AUUUA in *VRK1* 3′UTR important for *VRK1* mRNA translation. (**a**) Schematic diagrams of *VRK1* 3′UTR deletion constructs are shown. (**b**) Relative luciferase activity in A549 cells transfected with *VRK1* 3′UTR deleted constructs were determined by luciferase assay (unpaired two-tailed Student’s *t*-test, *n* = 4; n.s., not significant; *** *p* < 0.001). (**c**) Schematic diagrams showing biotinylated RNA fragments spanning from the beginning of 3′UTR of *VRK1* to the end, which were used for pull-down. (**d**) Biotinylated RNA fragments were incubated with cytoplasmic lysates from HEK293A cells; after biotinylated RNA fragments were pulled-down using streptavidin beads, the levels of HNRNP A1 bound to the biotinylated RNA segments were detected by immunoblot analysis.

The secondary structure and the sequence of 3′UTR are known to affect RNA localization, stability and translation. Thus, to determine the region on *VRK1* 3′UTR that is responsible for translation enhancement, we generated a predicted secondary structure of the *VRK1* 3′UTR using mfold software. *VRK1* 3′UTR showed three different regions containing loop domains, so we first generated a serially-deleted structure of the *VRK1* 3′UTR and inserted them in the reporter vector to examine the luciferase activity of the sequences (Figure 3a). The deletion of nucleotides 121–360 (1–120) significantly decreased translation activity while the deletion of 361–405 (1–360) still showed translation enhancement (Figure 3b). These results suggest that the region between 121 and 360 is absolutely required for translation activity in *VRK1* 3′UTR. To further verify the important sequence for HNRNP A1 binding, we confirmed HNRNP A1 binding affinity using a construct of fragmented *VRK1* 3′UTR (Figure 3c). We found a good interaction between HNRNP A1 and VRK1 with 111–230 constructs (Figure 3d). This is in agreement with our previous finding that the region between 121 and 360 in the *VRK1* 3′UTR acts as a *cis*-acting element, which we confirmed in the even narrower region of 121–230.

Given that hnRNP A1 has ability to bind to AUUUA-rich sequences [24,25], we analyzed the *VRK1* mRNA sequence to determine whether a region for HNRNP A1 association is present. Interestingly, we found that one region in the *VRK1* 3′UTR for HNRNP A1 association. The sequence is in the region between 121 and 360, which is thought to be a *cis*-acting region according to our previous experiments. Additionally, the phylogenetic analysis identified a highly conserved AUUUA sequence within the same region of the *VRK1* 3′UTR (Figure S2a). To experimentally check the role of this sequence in the translation activation of 3′UTR, we analyzed luciferase activity with the wild-type (WT) and mutation construct. The mutant reporter harboring a mutation of the AUUUA sequence in the *VRK1* 3′UTR showed slightly diminished activity compared with the WT *VRK1* 3′UTR (Figure S2b). Furthermore, we confirmed that HNRNP A1 was clearly bound to biotinylated WT *VRK1* 3′UTR. The interaction of HNRNP A1 with mutant *VRK1* 3′UTR was significantly lower than that with the WT (Figure S2c). HNRNP A1 was bound by biotinylated-*VRK1* 3′UTR more than *Nfil3* 5′UTR and this binding was reduced by competition with unlabeled-*VRK1* 3′UTR (Figure S2d). Taken together, we suggest that the secondary stem-loop containing a AUUUA sequence is a *cis*-acting element in *VRK1* 3′UTR-mediated translation.

### 2.4. RRM1, RRM2 and RGG Domain of HNRNP A1 Cooperatively Binds to the VRK1 mRNA

**Figure 4:**
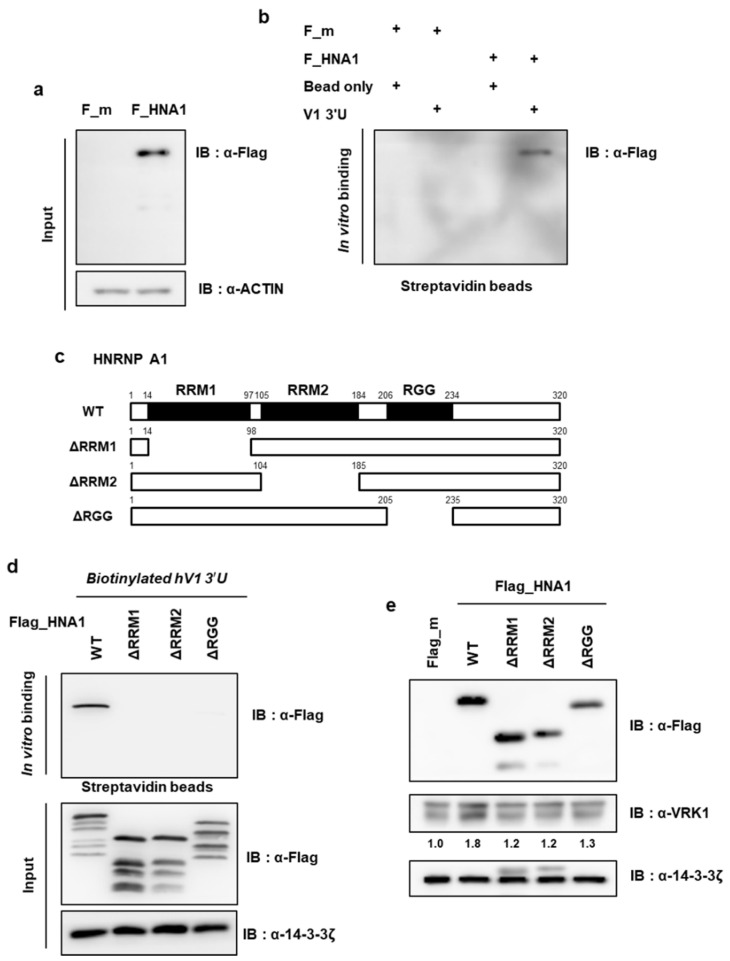
Mapping the interaction domains of HNRNP A1. (**a**,**b**) The in vitro-transcribed *VRK1* 3′UTR construct was labeled with biotin-UTP and was incubated with cell lysates of Flag_HNRNP A1 (F_HNA1)-overexpressing HEK293A. Biotin-UTP labelled *VRK1* 3′UTR was pulled down with streptavidin bead. Streptavidin affinity-purified samples were separated by SDS-PAGE and were subjected to immunoblot analysis with the indicated antibodies. (**c**) Schematic representation of WT HNRNP A1 and its truncation mutants is shown. (**d**) The domain of HNRNP A1 that interacts with the *VRK1* 3′UTR was mapped by creating truncations of HNRNP A1 tagged with Flag and by testing their interaction using biotin pull-down and immunoblot assay with anti-Flag antibody. (**e**) After the transfection of HEK293A cells with WT HNRNP A1 or its truncation mutants, the levels of VRK1 and loading control, 14-3-3ζ, were analyzed by western blot analysis.

HNRNP A1 is composed of three major domains: N-terminal RNA Recognition Motif (RRM) 1, RRM2 and the C-terminal RGG box region. The N-terminal region encompassing the RRMs (residues 1–196) of HNRNP A1 is referred to as unwinding protein 1 (UP1) and is the primary RNA-binding domain. UP1 shares a high degree of sequence homology, bringing the two β-sheet surfaces of hnRNP A1 closer together [26]. To verify whether RRM domains are also important for HNRNP A1 binding to *VRK1* mRNA, we conducted an in vitro binding assay using a series of mutant HNRNP A1 proteins in which each domain was deleted. After confirming that exogenous HNRNP A1 binds to *VRK1* 3′UTR (Figure 4a,b), the *VRK1* mRNA-interacting domain within HNRNP A1 was mapped using a series of HNRNP A1 truncation or deletion constructs (Figure 4c). Interestingly, all domains were necessary for HNRNP A1 and *VRK1* mRNA interaction, because when any one of the three domains was lost, the binding decreased (Figure 4d). We confirmed that the AUUUA consensus sequence was not the only factor needed for the binding of HNRNP A1; instead, the presence of the entire secondary structure containing the AUUUA sequence was required (Figure 3 and Figure S2). The reason why the interaction between HNRNP A1 and VRK1 was lost when each of the domains were lost may be due to the importance of the entire AUUUA-containing secondary structure, not just the AUUUA sequence. Consistently, ectopically expressed HNRNP A1, which is missing each domain, did not lead to the enhanced endogenous VRK1 protein levels seen with the WT (Figure 4e). Collectively, we conclude that the three domains of HNRNP A1 are important for binding to *VRK1* mRNA.

### 2.5. HNRNP A1 Interacts with Translation Initiation Factor EIF3B

**Figure 5:**
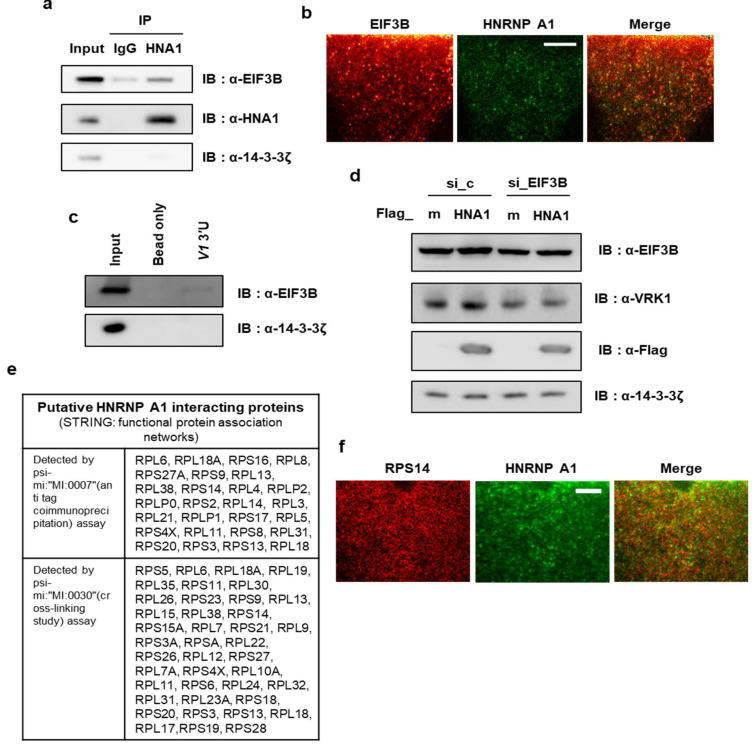
HNRNP A1 interacts with translation initiation factor EIF3B and ribosomal proteins. (**a**) HEK293A cells were subjected to immunoprecipitation with HNRNP A1. Immunoblotting was performed with specific antibodies. (**b**) The localization of HNRNP A1 and EIF3B was observed with a stimulated emission depletion (STED) fluorescence microscopy. Scale bar, 20 μm. (**c**) Biotin-RNA pulldown indicated that EIF3B was capable of binding with the 3′UTR of *VRK1* in HEK293A cells. (**d**) Either si_c or EIF3B targeting siRNAs (si_EIF3B) were transfected with either Flag_mock (F_m) or Flag_HNRNP A1 (F_HNA1) into HEK293A cells. After 36 h incubation, cells were subjected to immunoblotting with annotated antibodies. (**e**) The table for putative HNRNP A1 interacting proteins identified by in silico prediction is shown. (**f**) Immunostaining was performed with HNRNP A1- or RPS14-specific antibodies. RPS14 was visualized using Alexa 594-conjugated secondary antibody. For HNRNP A1, Alexa 647-conjugated secondary antibody was used for visualization with a STED microscopy. Scale bar, 20 μm.

Next, we wanted to understand how HNRNP A1 enhances the translation of *VRK1* mRNA in a 3′UTR-dependent manner. A previous report showed that the interaction of eIF4G with poly (A)-binding protein (PABP) results in mRNA circularization and stimulates translation [27]. This conformation of the mRNA is thought to promote efficient translation, probably by accelerating ribosome recycling. Thus, we hypothesized that 3′UTR-bound HNRNP A1 recruits translation-related factors, which represent a significant molecular feature in the regulation of efficient translation. Recent work has shown that the RNA-binding protein AUF1 on the 3′UTR of *Cry1* mRNA recruits the 40S ribosomal subunit to the 5′ end of the mRNA by associating with EIF3B, leading to CRY1 expression [28]. To verify the interaction between HNRNP A1 and EIF3B, endogenous EIF3B was immunoprecipitated using a specific antibody for HNRNP A1. Interestingly, EIF3B was co-immunoprecipitated with HNRNP A1 (Figure 5a), indicating that HNRNP A1 can associate with EIF3B through protein–protein interactions. Additionally, we visualized the cellular interaction and colocalization of HNRNP A1 with EIF3B using stimulated emission depletion (STED) fluorescence microscopy. HNRNP A1 was detected to be co-localized with EIF3B (Figure 5b) in the cytoplasm. When we checked the binding of EIF3B to the *VRK1* 3′UTR construct, EIF3B appeared to bind with the full-length *VRK1* 3′UTR (Figure 5c). Furthermore, while VRK1 protein levels were increased by the overexpression of HNRNP A1, this increase was prevented by silencing EIF3B (Figure 5d). From these results, we suggest that HNRNP A1 is involved in the recruitment of the translation machinery and that the 3′UTR-mediated translation activation of *VRK1* is mediated by EIF3B binding.

To more objectively and comprehensively identify HNRNP A1-binding partners related to the translational process, we conducted an in silico analysis using the STRING database. From two independent experiments using the STRING and BioGRID databases, we identified many several ribosomal proteins, particularly 40S ribosomal proteins, as HNRNP A1-binding proteins (Figure 5e and Figure S3a). To verify the interaction between HNRNP A1 and 40S ribosomal proteins identified with the in silico analysis, we performed immunocytochemistry and confirmed that RPS14 was co-localized with the HNRNP A1 protein (Figure 5f). Taken together, these results suggest that 3′UTR-bound HNRNP A1 recruits EIF3B and 40S ribosomal proteins and that these interactions may activate 3′UTR-mediated translation of *VRK1* mRNA.

### 2.6. HNRNP A1 Promotes Lung Cancer Cell Proliferation and Cell Cycle Progression

**Figure 6:**
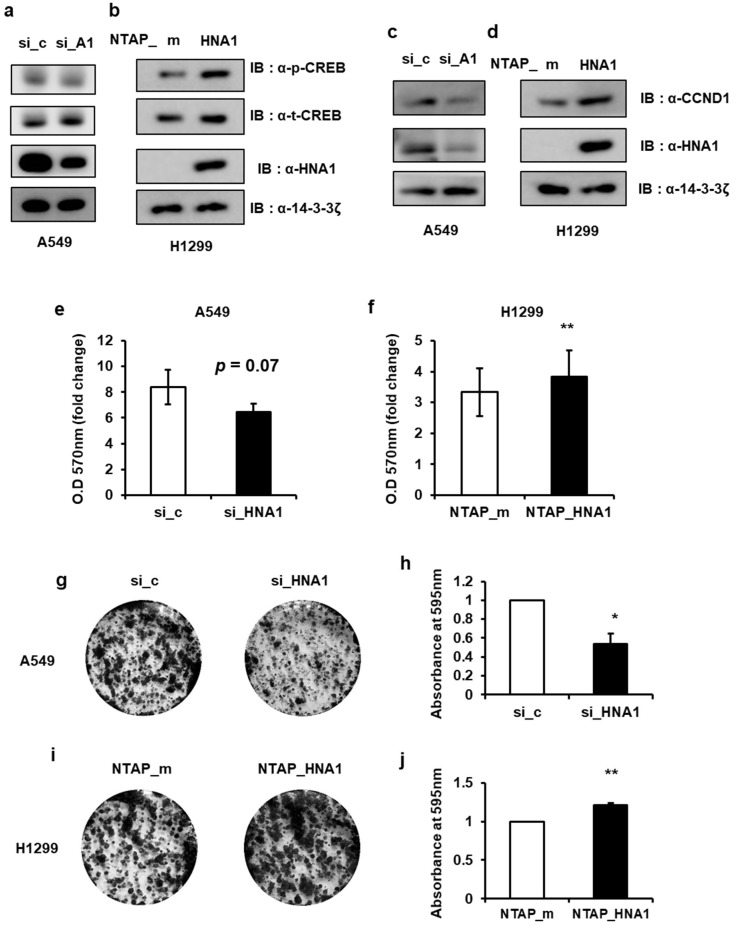
HNRNP A1 regulates lung cancer cell proliferation. (**a**,**c**) A549 cells were transfected with either si_con (si_c) or si_HNRNP A1 (si_HNA1). Cells were immunoblotted with the indicated antibodies. (**b**,**d**) Immunoblotting of cell lines stably expressing NTAP_HNRNP A1 (NTAP_HNA1) was performed. Western blot analysis was performed with the indicated antibodies. (**e**,**f**) Cell proliferation of cells after HNRNP A1 silencing or of NTAP_HNRNP A1 stably expressing cells were assessed with MTT assays for 5 days after seeding. Cell proliferation (%) were calculated relative to that of at the time of transfection, which was set to 100%. (**g**–**j**) Colony formation assays were performed. Cells were grown for 10 days and were stained with crystal violet (left). Colonies were quantified by measuring the absorbance of extracted crystal violet at 595 nm (right) (* *p* < 0.05, ** *p* < 0.01).

Our findings indicate that the binding of HNRNP A1 to the 3′UTR of *VRK1* mRNA enhances its translation. To examine the effect of HNRNP A1 in lung tumorigenesis, we assessed the effect of HNRNP A1 in tumor cell proliferation and tumor progression. As a serine/threonine kinase, VRK1 promotes cell cycle progression through the phosphorylation of various substrates, such as p53 at Thr18, CREB at Ser133, histone H3 at Thr3 and Ser10 and BAF at Ser4, during each phase of the cell cycle. The loss of HNRNP A1 or VRK1 results in a delay of cell cycle progression in the G1/S transition. Therefore, we employed CREB, a representative VRK1 substrate, to assess the effect of HNRNP A1 on VRK1 expression. We showed that the depletion of HNRNP A1 inhibits the VRK1-mediated phosphorylation of CREB in A549 lung cancer cell line (Figure 6a). Conversely, HNRNP A1 overexpression enhanced CREB phosphorylation in H1299 cells that originated from lung cancer (Figure 6b). CREB is a transcription factor that promotes expression of cyclin D1 (CCND1), which forms a complex with CDK4/6 and is required for the cell cycle during the G1/S transition. Consistent with previous results, the siRNA-mediated knockdown of HNRNP A1 led to a decrease in CCND1 levels; however, CCND1 levels increased in H1299 cells with stable HNRNP A1 expression (Figure 6c,d).

We determined the effect of HNRNP A1 on tumor cell proliferation using MTT and colony forming assays. In lung cancer cell lines, cell proliferation decreased in cells with knockdown HNRNP A1 but increased in HNRNP A1 stably expressing cells (Figure 6e,f). In both cells, the rate of colony formation was also assessed. Cells with knockdown HNRNP A1 exhibited a lower rate of colony formation than the control cells (Figure 6g,h); however, this rate was higher in HNRNP A1 stably expressing cells (Figure 6i,j). Together, our findings indicate that HNRNP A1 regulates CREB phosphorylation and enhances lung cancer cell proliferation.

### 2.7. Targeting VRK1 3′UTR Affects VRK1 mRNA Translation and Inhibits Lung Cancer Cell Proliferation

We observed the effects of HNRNP A1 in vitro through a series of VRK1 gene expression and functional assays. To further see whether the effects of regulation by HNRNP A1 in lung cancer cells proliferation were obtained through *VRK1* mRNA translation and more precisely in a 3′UTR-dependent manner, we generated a genome-edited A549 cell line lacking the *cis*-acting region of VRK1 3′UTR (Figure 3a). This enabled us to confirm the translational regulation of endogenous *VRK1* 3′UTR by HNRNP A1 and examine the VRK1-mediated role of HNRNP A1 in lung cancer cells. As mentioned previously, we confirmed that the binding of HNRNP A1 to *VRK1* 3′UTR was mediated by a stem-loop containing the AUUUA consensus sequence because the binding region required the interaction with the entire *cis*-acting element (Figure 3 and Figure S2) and the domains of the *trans*-acting factor (Figure 4).

**Figure 7:**
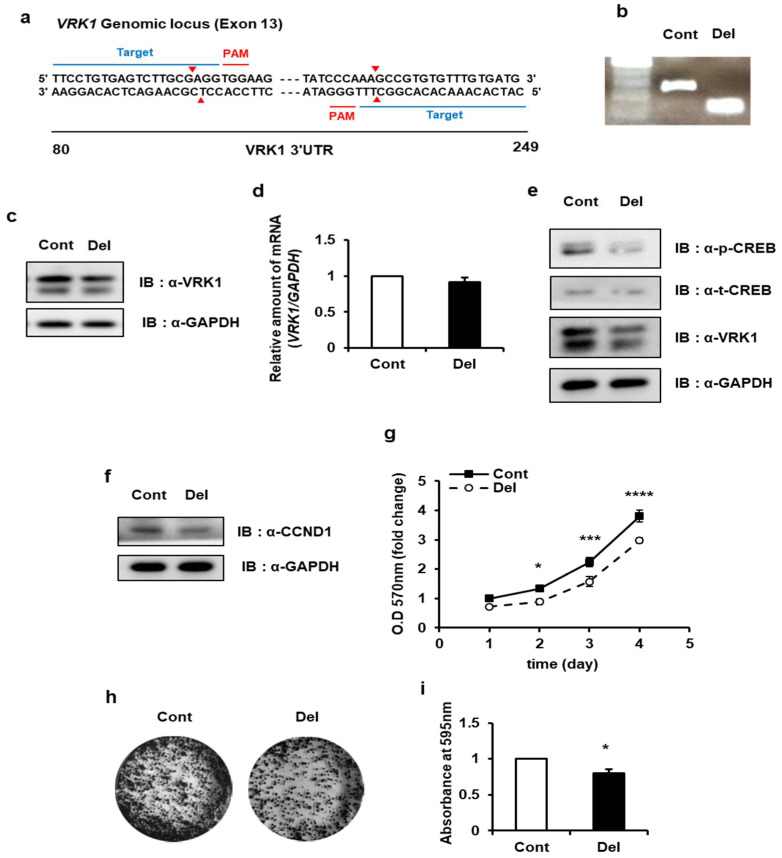
CRISPR/Cas9-mediated deletion of *cis*-acting element in *VRK1* 3′ UTR suppresses lung cancer cells growth. (**a**,**b**) A deletion of *cis*-acting region in *VRK1* 3′ UTR of lung cancer cell line was generated using the CRISPR/Cas9 system. The genome-edited cell line was selected by single cell sorting. Blue bars indicate the location of paired sgRNAs. Red arrowheads show expected deletion of the genomic DNA. Genotyping analysis identified an A549 cell line lacking the *cis*-acting region. (**c**) VRK1 protein expression was effectively decreased in the genome-edited cell line compared to the control. (**d**) qRT-PCR for *VRK1* mRNA transcripts in the genome-edited cell line showed unchanged *VRK1* mRNA levels. (**e**,**f**) p-CREB and CCND1 protein levels were decreased in *cis*-acting element deleted cells. (**g**) Cell proliferation was tested with MTT. Measurement of MTT conversion by absorbance at 570 nm showed that there was decreased proliferation with *cis*-acting region deleted cells (Two-way analysis of ANOVA with Sidak’s multiple comparisons, *n* = 4, * *p* < 0.05, *** *p* < 0.001, **** *p* < 0.0001). (**h**,**i**) Colony formation assays were performed in 6-well plates. Cells were grown for 10 days and were stained with crystal violet (left). Colonies were quantified by measuring the absorbance of extracted crystal violet at 595 nm (right) (Unpaired two-tailed *t*-test, *n* = 4, * *p* < 0.05).

Thus, we designed gRNA targeting each side of PAM sequences of the *cis*-acting element in *VRK1* 3′UTR (Figure 7a). After single cell selection by sorting, we first identified a cell line that had the specific deletion of the *cis*-acting region of *VRK1* 3′UTR in the genome (Figure 7b). In this genome-edited cell line, VRK1 protein expression was significantly decreased (Figure 7c), even though the amount of *VRK1* mRNA transcripts showed no change (Figure 7d). Together, our results suggest that VRK1 expression in A549 cells is indeed regulated by HNRNP A1-dependent and *VRK1* 3′UTR-mediated translation.

To further support the role of HNRNP A1 in *VRK1* 3′UTR-mediated translation of lung cancer cells, we wanted to test the properties of the cell line with the *cis*-acting region deleted as we have done previously (Figure 6). We examined the phosphorylation levels of CREB and the total levels of CCND1 by Western blot analysis and found that both levels were decreased in genome-edited cells compared with the controls, which agreed with our perspective (Figure 7e,f). Consistent with the effects of HNRNP A1 siRNAs on cell proliferation, cells without the *cis*-acting region showed significantly inhibited cell proliferation. MTT assays revealed that the growth was suppressed in cells without the *cis*-acting region (Figure 7g). As shown in Figure 7h,i, the relative colony formation efficiency was also significantly reduced in these cells. To understand the effect of HNRNP A1 in VRK1-mediated cell cycle progression of lung cancer cells, we performed FACS analysis in cells without the *cis*-acting region. The number of G1 phase-gated cells was significantly higher in the *cis*-acting region deleted cells than the control cells (Figure S4a).

We confirmed that HNRNP A1 cooperatively binds to *VRK1* 3′UTR through its RRM1, RRM2 and RGG domains. The architecture and organization of the two RRMs are known to be essential to hnRNP A1 function [29]. Additionally, the RGG/RG domain, which is present in HNRNP A1, is prevalent throughout eukaryotes and is the second most common RNA-binding domain in the human genome. The RGG/RG domain is found in several other RNA-binding proteins such as FUS, FMRP and HNRNP U and it has been proposed as an RNA-binding motif. Furthermore, strong synergistic binding between the RRM and adjacent RGG/RG domains is required to achieve RNA-binding affinities of the full-length FUS. Thus, to distinguish the role of HNRNP A1 on *VRK1* mRNA, we made cells with the RGG domain of HNRNP A1 deleted using Crispr/Cas9 system. This deletion resulted in low levels of VRK1 protein and phosphorylated CREB and, subsequently, suppressed lung cancer cell proliferation (Figure S4b–e). Therefore, these results indicate that HNRNP A1 has an important role in lung cancer cell proliferation through *VRK1* 3′UTR-mediated translation.

### 2.8. HNRNP A1 Is Upregulated in Human Lung Tissues

**Figure 8:**
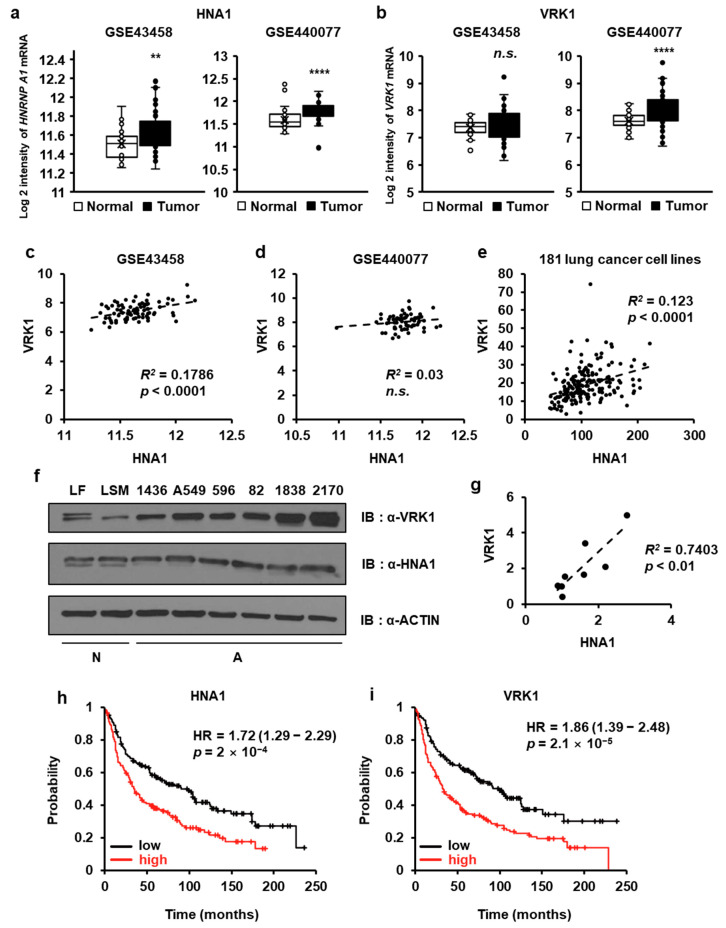
*HNRNP A1* is upregulated in lung cancer. (**a**,**b**) Analysis of publicly available TCGA lung cancer data sets revealed a significant up-regulation with *HNRNP A1* expression but not with *VRK1* expression in lung cancer. ** *p* < 0.01, **** *p* < 0.0001, n.s., not significant. (**c**–**e**) Co-expression analysis between *HNRNP A1* and *VRK1* in different lung cancer data sets are shown. (**f**) Cell lysates of human normal lung cells (lung fibroblast (LF), lung smooth muscle (LSM)) and a panel of lung cancer cell lines (NCIH1436 (1436), A549, NCIH596 (596), NCIH82 (82), NCIH1838 (1838), NCIH2170 (2170)) were probed for the expression of VRK1 and HNRNP A1 using specific antibodies. N, normal; A, adenocarcinoma. (**g**) Band intensities were measured by densitometry using Image J. VRK1 and HNRNP A1 band intensities were normalized to those of ACTIN. Co-expression analysis between HNRNP A1 and VRK1 protein levels is shown. (**h**,**i**) Online survival analysis software (Kaplan-Meier plotter) was used to assess the prognostic value of *HNRNP A1* and *VRK1* expression in lung cancer patient. *HNRNP A1* and *VRK1* overexpression correlates with poor prognosis of lung cancer patients.

To evaluate the role of HNRNP A1 in lung oncogenesis, we first performed gene profiling analysis in lung adenocarcinomas and normal lung tissues (GEO accession: GSE43458 and GSE440077) from publicly available gene expression datasets. This analysis revealed that the expression of *HNRNP A1* mRNA was upregulated in adenocarcinomas relative to control tissues (Figure 8a). Given that the level of VRK1 is upregulated in several cancer cells and tissues, we checked whether *VRK1* is upregulated at the transcript level. The levels of *VRK1* mRNA were significantly upregulated in the GSE440077 dataset but not in GSE43458 dataset (Figure 8b). These results showed that upregulated *VRK1* at the transcript level in lung cancer tissues is not a common phenomenon. Instead, the levels of VRK1 proteins and the mechanisms behind its post-transcriptional regulation are more important.

HNRNP A1 is an RNA-binding protein that governs RNA splicing or mRNA degradation of several target genes [30,31,32]. To confirm whether HNRNP A1 regulates *VRK1* mRNA translation and not mRNA stability, we examined data on the co-expression between *HNRNP A1* and *VRK1* from TCGA. In GSE43458 and 181 lung cancer cells, a weak positive correlation was found between *HNRNP A1* and *VRK1* mRNA expression. Furthermore, no relationship between two genes was observed in the GSE440077 dataset (Figure 8c–e). To corroborate our TCGA findings, we next looked for the expression of HNRNP A1 and VRK1 in normal lung cell lines (lung fibroblast cell line (LF) and lung smooth muscle (LSM)) and lung cancer cell lines via immunoblotting. Consistent with our TCGA findings, HNRNP A1 and VRK1 expression was significantly upregulated in lung cancer cells compared with normal cells and the expression of HNRNP A1 and VRK1 was positively correlated (*R^2^* = 0.7403, *p* < 0.01, Figure 8f,g). Additionally, we performed a Kaplan-Meier survival analysis using the Kaplan-Meier plotter, an online tool. Our analyses indicated that higher *HNRNP A1* and *VRK1* mRNA expression was correlated with the poor overall survival of patients with lung cancer (Figure 8h,i). Altogether, our transcriptome analyses demonstrated that *HNRNP A1* mRNA expression is enriched in lung tumors and that the level of HNRNP A1 or VRK1 is correlated with lung cancer patient survival.

## 3. Discussion

The mutation or aberrant expression of a gene involved in the cell cycle may result in human diseases, including cancer. Recent findings suggest that post-transcriptional control of an mRNA is critical for controlling gene expression, with many considered to be the primary culprits of various disorders. Different classes of RNA-binding proteins (RBPs), including heterogeneous nuclear RNPs (hnRNPs), serine–arginine RBPs (SR RBPs) and miRNAs, are known to mediate the post-transcriptional control of mRNA. To date, more than 1500 RBPs have been identified in humans (7.5% of the proteome) [33] and these proteins contribute quantitatively and qualitatively to the protein profile of a cell. However, RBPs possess both tumor suppressive and oncogenic functions. Therefore, a great deal of research is needed to understand why and how they influence the development and the progression of cancers.

HNRNP A1 is a member of the A/B subfamily of ubiquitously expressed HNRNPs, which have a wide variety of functions. Recently, HNRNP A1 was found to be overexpressed in several cancer types, including lung, breast and colon cancer. One study showed that *HNRNP A1* mRNA was higher in SCLC than in NSCLC [16]. Additional reports have suggested that HNRNP A1 may promote cancer cell proliferation and tumor angiogenesis. However, the role of HNRNP A1 in cancer progression has not been clearly defined. The objective of this study was to determine whether HNRNP A1 is a post-transcriptional driver of cancer progression. In this report, we demonstrated the biological functions and mechanisms of HNRNP A1 using lung cancer cell lines with the knockdown or stable expression of HNRNP A1. The knockdown of HNRNP A1 reduced cell proliferation while the overexpression of HNRNP A1 led to increased growth in lung cancer cells, establishing HNRNP A1 as a growth stimulator (Figure 6).

Serine/threonine protein kinase VRK1 plays a regulatory role in the cell cycle, being involved key cell cycle events such as G0 exit and entry into G1 [34], chromatin compaction in G2/M [19] and regulation of nuclear envelope assembly and disassembly [35]. The overexpression or amplification of VRK1 has been observed in several types of tumors, implying that it contributes to tumorigenesis [20,36]. However, until now, the regulation of *VRK1* gene expression has not been studied in cancer. Herein, we report that VRK1 is post-transcriptionally upregulated in lung cancer cells, leading to the increased expression of CCND1. *VRK1* 3′UTR upregulates VRK1 expression by enhancing mRNA translation (Figure 1). We found that HNRNP A1 is associated with a 3′UTR of *VRK1* mRNA and modulating HNRNP A1′s abundance did not alter *VRK1* mRNA levels but did regulate 3′UTR-mediated *VRK1* mRNA translation (Figure 1 and Figure 2). Further subdivision of the 3′UTR revealed that the secondary structure containing the AUUUA sequence, spanning from 121 to 230, is important in translation activation and specifically interacts with HNRNP A1 (Figure 3 and Figure S2). To further clarify the functions of HNRNP A1 in *VRK1* mRNA regulation, we established a cell line with a deletion of the *VRK1* 3′UTR *cis*-acting element using CRISPR/CAS9 technology. Importantly, only CRISPR/CAS9-mediated deletion of the *cis*-acting element on *VRK1* 3′UTR decreased VRK1 protein levels, resulting in a reduction of lung cancer cell proliferation. This suggests that *VRK1* 3′UTR is important for the enhancement of VRK1 protein levels and that HNRNP A1 regulates lung cancer cell progression via *VRK1* 3′UTR-mediated translational regulation (Figure 7). Furthermore, we discovered that the *trans*-acting domains of HNRNP A1 affect the translational regulation of VRK1. More interestingly, a clear effect of HNRNP A1-mediated post-transcriptional regulation of VRK1 was clarified through relevant phenotypes of the CRISPR/Cas9-mediated mutant of HNRNP A1 (Figure 4 and Figure S4).

To elucidate the role of HNRNP A1 in translation, we investigated HNRNP A1-interacting proteins and found that HNRNP A1 may interact with translation initiation factors, particularly EIF3B and several ribosomal proteins (Figure 5 and Figure S3). Circularization of mRNA could facilitate a direct recycling of ribosomes or ribosomal subunits after termination at the stop codon, resulting in the synergistic enhancement of translation. The interaction between HNRNP A1 and translation initiation factor EIF3B may circularize mRNAs and subsequently accelerate translation efficiency. Furthermore, EIF3B is one of the 104 proteins participating in the 3′UTR-mediated translational regulation pathway, as identified by the Harmonizome: a collection of processed datasets gathered to serve and mine knowledge about genes and proteins from over 70 major online resources [37]. Using http://kmplot.com/analysis (accessed on 15 January 2018), we also assessed the correlation between EIF3B overexpression and clinical prognosis (Figure S5). The present study is the first report to reveal the functional role of HNRNP A1 in 3′UTR-mediated translation.

The expression of *HNRNP A1* and *VRK1* at the transcripts level was measured using publicly available datasets of lung cancer tissues compared with non-tumor tissues (Figure 8). We found that *HNRNP A1* was significantly overexpressed in tumors compared with normal tissues, but the upregulation of *VRK1* expression in cancer was contradictory. These data indicate that post-transcriptional regulation of the VRK1 is also important for gene expression pattern in cancer. We further investigated the relationship between *HNRNP A1* and *VRK1* in lung cancer samples and lung cancer cell lines. Scatter plot analyses revealed a weak correlation between *HNRNP A1* and *VRK1* mRNA levels in lung cancer tissues; however, lung cancer cells had significantly higher HNRNP A1 and VRK1 protein expression than normal lung cells. Furthermore, a significant positive correlation was found between HNRNP A1 and VRK1 protein levels. Importantly, a correlation was found between *HNRNP A1* or *VRK1* expression and poor prognosis for patients with lung cancer.

Altogether, our findings identified that VRK1, a key regulator of lung cancer cell proliferation, is a translation target of HNRNP A1. Importantly, the existence of translation enhancement in the 3′UTR of *VRK1* mRNA may have a significant meaning in the differential patterns of VRK1 gene expression in cancer cells. We report that HNRNP A1, a novel pro-tumorigenic RBP in lung cancer, contributes to lung cancer cell proliferation by promoting the expression of the oncogene *VRK1* by binding to 3′UTR of *VRK1* mRNA, leading to enhanced *VRK1* mRNA translation in lung cancer cells. The functional relevance of HNRNP A1 in cell cycling and the enhanced expression of HNRNP A1 in lung cancer strongly imply that HNRNP A1 is involved in lung carcinogenesis. The expression levels of HNRNP A1 alone or in combination with VRK1 in patients with lung cancer are important because they provide not only a predictor for lung cancer prognosis but also a potential therapeutic target in lung cancer.

## 4. Materials and Methods

### 4.1. Plasmid Constructs

To generate psiCHECK2 VRK1 3′UTR, human VRK1 (accession No. NM_003384.3) 3′UTR was amplified using pfu polymerase (SolGent, Daejeon, Korea) with specific primers and the sequence was confirmed by sequencing. To generate serially-deleted VRK1 3′UTRs constructs, VRK1 3′UTR were amplified using pfu polymerase (SolGent) with specific primers from full-length human VRK1 cDNA. For the in vitro binding analysis, full-length and the fragments of the VRK1 3′UTRs were amplified as previously described. PCR products were digested with specific enzymes of EcoRI/XbaI (Beams Bio, Seongnam-si, Korea) and were subcloned into pBluescript SK(+) (pSK (kindly gifted by Dr. Sung Key Jang from POSTECH)) to generate pSK-VRK1 3′UTR.

In order to construct plasmids for CRISPR-mediated gene editing, PX458 and PX459 vectors were used as a backbone [38]. pSpCas9(BB)-2A-GFP (PX458) and pSpCas9(BB)-2A-Puro (PX459) were a gift from Feng Zhang (Addgene plasmid # 48,138; http://n2t.net/addgene:48138 (accessed on 15 January 2018); RRID:Addgene_48138 for PX458 and Addgene plasmid # 62,988; http://n2t.net/addgene:62988 (accessed on 15 January 2018); RRID:Addgene_62988 for PX459). A 25mer of the selected sequence of sgRNAs (sgRNA-top: 5′-CACCGTTCCTGTGAGTCTTGCGAGG-3′, sgRNA-bottom: 5′-AAACCCTCGCAAGACTCACAGGAAC-3′ and sgRNA-top: 5′-CACCGCATCACAAACACACGGCTTT-3′, sgRNA-bottom: 5′-AAACAAAGCCGTGTGTTTGTGATGC-3′) targeting DNA region within 3′UTR of VRK1 and separate sgRNAs (sgRNA-top: 5′-CACCGATGACAACTTCGGTCGTGG-3′, sgRNA-bottom: 5′-AAACCCACGACCGAAGTTGTCATC-3′) targeting DNA region within RGG domain of HNRNP A1 were selected from in silico tools that predicts PAM target sites.

### 4.2. Cell Culture and Drug Treatment

A549, H1299 and HEK293A cells were grown in Dulbecco’s modified Eagle’s medium supplemented with 10% heat-inactivated fetal bovine serum (FBS) and 100 U/mL of penicillin G and streptomycin. All cell lines were authenticated prior to experiments and they are not listed as commonly misidentified cell lines by the International Cell Line Authentication Committee (ICLAC). Cell identity and status were regularly checked. To block the transcription, cells were treated with 5 μg/mL actinomycin D (Sigma, Cat.# A9415) and then harvested at the indicated time points.

### 4.3. Generation of Stable Cell Lines

Generation of H1299 stable cells expressing HNRNP A1 was performed as previously described. Briefly, pNTAP_mock or pNTAP_HNRNP A1 vector was transfected into the H1299 cell line using Lipofectamine 2000 (Invitrogen, Carlsbad, CA, USA) and were incubated for 24 h. Then, cells were selected by 800 μg/mL G418 with media changes every 2 days until single colonies were formed.

To generate HNRNP A1 RGG domain- or VRK1 3′UTR *cis*-acting element- deleted cells, A549 cells were cultured in 6-well dishes to 70% confluence and either PX458 or PX459 plasmid was introduced to A549 cells using Lipofectamine 2000. After 48 h incubation, cells transfected with PX459 for RGG domain deletion were selected by 5 μg/mL puromycin. Puromycin-resistant cells were maintained in DMEM supplemented with 10% fetal bovine serum, 1% penicillin–streptomycin and 2 μg/mL puromycin. Single colonies of RGG domain-deleted cells or GFP-positive, *cis*-acting element-deleted cells were suspended and were sorted using a Mo-Flo XDP (Beckman Coulter, Miami, FL, USA).

### 4.4. Transient Transfection and RNA Interference

Plasmid expression or siRNA transfection for transient knockdown in A549 or H1299 was performed using Lipofectamine 2000 (Invitrogen, Carlsbad, CA, USA) or Microporator MP-100 (Digital Bio, Seoul, Korea) according to manufacturer’s recommendations. The sequences of siRNAs were as follows. si_c: 5′-UUCUCCGAACGUGUCACGUTT-3′, si_HNA1: 5′-GGACUGUAUUUGUGACUAATT-3′, si_EIF3B: 5′-GAGUAUGAACGGUGCCUUA-3′. Synthesized siRNAs were purchased from Bioneer (Bioneer, Daejeon, Korea).

### 4.5. MTT Assay and Colony Formation Assay

The MTT assay assessed proliferation of A549 and H1299 cells. Cells were plated in a 96-well plate with the density of 5 × 10^3^ cells per well and were cultured for 12 h in growth media. After every 24 h, 25 μL MTT solution was added to the cells (5 mg/mL; Sigma-Aldrich, St. Louise, MO, USA) along with 25 μL growth media and cells were incubated at 37 °C for 2 h. Culture media and MTT solution were then removed and 100 μL DMSO was added to each well. Crystals of MTT-formazan were dissolved at 37 °C for 4 h. Absorbance was measured on a microplate reader (Tecan, Männedorf, Switzerland) at 570 nm.

For colony formation assay, cells were seeded at a density of 2 × 10^3^ cells per well in 6-well dishes. Six days after seeding, cells were stained with 0.5% crystal violet in 25% methanol for 1 h. Plates were then washed with PBS to remove excessive dye and were photographed with a digital camera (Canon, Japan). Quantitative changes in clonogenicity were determined by extracting colonies with 20% acetic acid and measuring the absorbance at 595 nm.

### 4.6. Dual Luciferase Reporter Assay

For the reporter assay, A549 or H1299 cells transfected with reporter or mock plasmids were lysed in Reporter Lysis 5X buffer (Promega, Madison, WI, USA). Renilla and firefly luciferase activities were determined by using the Dual-Luciferase^®^ Reporter Assay System (Promega, Madison, WI, USA) according to the manufacturer’s instructions.

### 4.7. Flow Cytometry Analysis of Cell Cycle

For flow cytometric analysis, the cells were trypsinized and then cell suspension was prepared in 1 mL PBS containing 1% BSA. Cells were fixed with 70% ethanol with 0.5% Tween-20 for O/N and then stained with propidium iodide (PI) solution (50 ug/mL PI and 100 ug/mL RNase A in PBS) for 30 min at room temperature. Samples containing 20,000 cells were then analyzed on a LSR Fortessa (BD Biosciences, San Jose, CA, USA).

### 4.8. RNA-Immunoprecipitation (RNA-IP)

HEK293A cells were lysed with RNA-IP buffer (10 mM HEPES (pH 7.5), 100 mM KCl, 5 mM MgCl_2_, 0.1% NP-40, protease inhibitor (Thermo scientific, Cat.# A32953)). Mouse IgG or HNRNP A1 antibody was incubated with HEK293A cell lysates at 4 °C overnight and then was incubated with Protein G beads at 4 °C for 4 h. We washed the beads 3 times with RNA-IP buffer and isolated RNAs using TRI reagent (Virginia Tech Bio-Technology, Cat.# TS200-001). RNA levels were quantified by qRT-PCR.

### 4.9. RNA Isolation and cDNA Synthesis

RNA isolation and cDNA synthesis were performed as we previously described [39]. Cells were lysed with TRI-Solution (Bio Science Technology, Cat.# TS100-001) and the total RNA was extracted. The yield and purity of RNA were determined by a NanoDrop 2000 spectrophotometer (Thermo Fisher Scientific, Waltham, MA, USA). Following quantification, 1 μg of each total RNA sample was reverse transcribed using oligo-dT and the ImProm-II Reverse Transcription System (Promega, Madison, WI, USA), according to the manufacturer’s instructions.

### 4.10. Quantitative Real-Time PCR

The mRNA levels of endogenous genes were detected by qRT-PCR using a StepOnePlus Real-Time PCR System (Applied Biosystems, Foster City, CA, USA) with the FastStart Universal SYBR Green Master (Roche, Mannheim, Germany) according to the manufacturer’s instructions. The following amplification program was used: polymerase activation at 95 °C for 10 min; 40 repeated cycles of 95 °C for 15  s and 60 °C for 1 min. The sequences of the forward and reverse primers are as follows: VRK1, 5′-AGACCCCAAAAGATGTCACG-3′ and 5′-CCAAGGAAGATGGCCAGTAA-3′ and GAPDH, 5′-GCCATCAATGACCCCTTCATT-3′ and 5′-GCTCCTGGAAGATGGTGATGG-3′.

### 4.11. In Vitro RNA Synthesis and In Vitro Binding Assay

For in vitro binding assays, biotin-UTP–labeled RNA was transcribed from the XbaI-linearized pSK-VRK1 3′UTR plasmids using T7 RNA polymerase (Promega, Madison, WI, USA). Streptavidin–biotin RNA affinity purification was performed as described previously [40]. In brief, cell extracts prepared from HEK293A cells were incubated with biotinylated-*VRK1* 3′UTR RNA and were subjected to streptavidin resin adsorption. Resin-bound proteins were analyzed by SDS–PAGE.

### 4.12. Immunoblot Analysis

Cells were lysed with RIPA buffer that contain protease inhibitor tablet (Thermo Scientific, Cat.# A32953), followed by sonication. Protein concentration of lysates were determined using Bradford reagent (AMRESCO, Solon, OH, USA). Proteins were resolved by SDS-PAGE and transferred to nitrocellulose membranes (Pall corporation, Port Washington, NY, USA), which were later incubated with blocking buffer (5% non-fat dry milk in Tris-buffered saline and 0.1% Tween 20) for 60 min. Horseradish peroxidase (HRP)-conjugated mouse (Thermo Scientific, Waltham, MA, USA, Cat.# 31430), rabbit (Promega), rat or goat (Bethyl Laboratories, Montgomery, TX, USA) secondary antibodies were detected with SUPEX ECL reagent (Neuronex, Daegu, Korea) and a LAS-4000 system (Fujifilm, Tokyo, Japan), according to the manufacturer’s instructions. Acquired images were analyzed using Image Gauge (Fujifilm) according to the manufacturer’s instructions. The integrated blot density was quantified through Image J software-based analysis (http://rsb.info.nih.gov/ij/ (accessed on 31 July 2018)).

### 4.13. Immunocytochemistry

Cells were maintained for 24 h, fixed with 4% paraformaldehyde for 20 min and were immunostained with appropriate primary and fluorescence-conjugated secondary antibodies. Coverslips were mounted onto slides using Fluoromount (Sigma-Aldrich, St. Louis, MO, USA). All images were obtained using a laser scanning confocal microscope (model FV1000; OLYMPUS) and FV10-ASW2.0 fluoviewer software was used for image analysis.

### 4.14. Statistical Analysis

All quantitative data are shown as the mean ± standard error of the mean (s.e.m.). Statistical analyses were performed using Student’s *t*-test, one-way analysis of variance (ANOVA) or two-way ANOVA and post-hoc Tukey’s multiple comparison tests or Sidak’s multiple comparisons using GraphPad software. *p* values of less than 0.05 were considered statistically significant. * *p* < 0.05, ** *p* < 0.01, *** *p* < 0.001, **** *p* < 0.0001.

## Data Availability

Not applicable.

## Supplementary Materials

The following are available online at https://www.mdpi.com/article/10.3390/ijms22115506/s1.
