# Supplementary material for: HNRNP A1 Promotes Lung Cancer Cell Proliferation by Modulating VRK1 Translation
Source: Int J Mol Sci. 2021 May 23;22(11):5506. doi: 10.3390/ijms22115506 (PMC8197126; doi:10.3390/ijms22115506)
Supplement: Supplementary file 1 [file ijms-22-05506-s001.zip › ijms-1162963-supplementary.pdf]

Figure S1. Ryu *et al.*

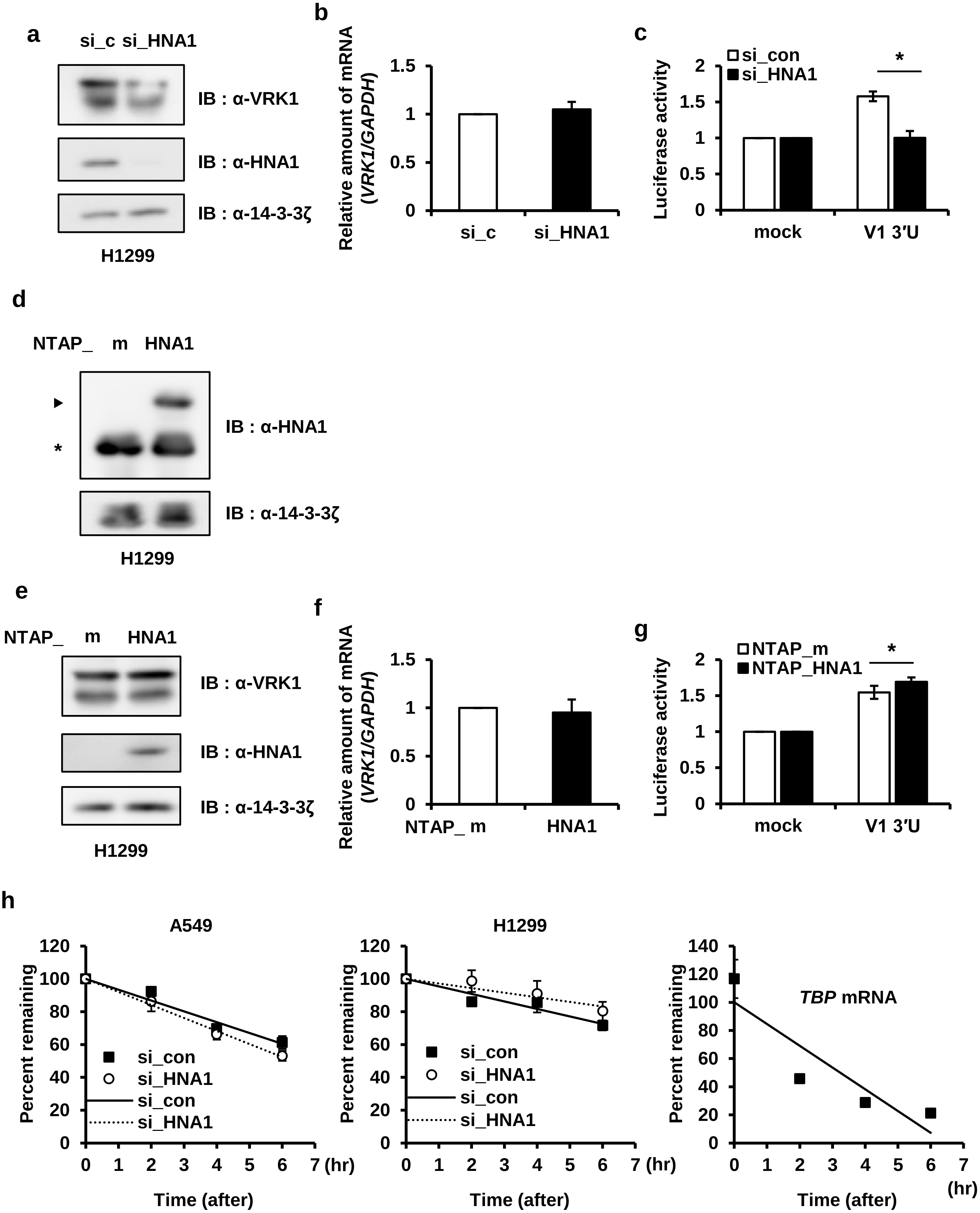

Figure S2. Ryu *et al.*

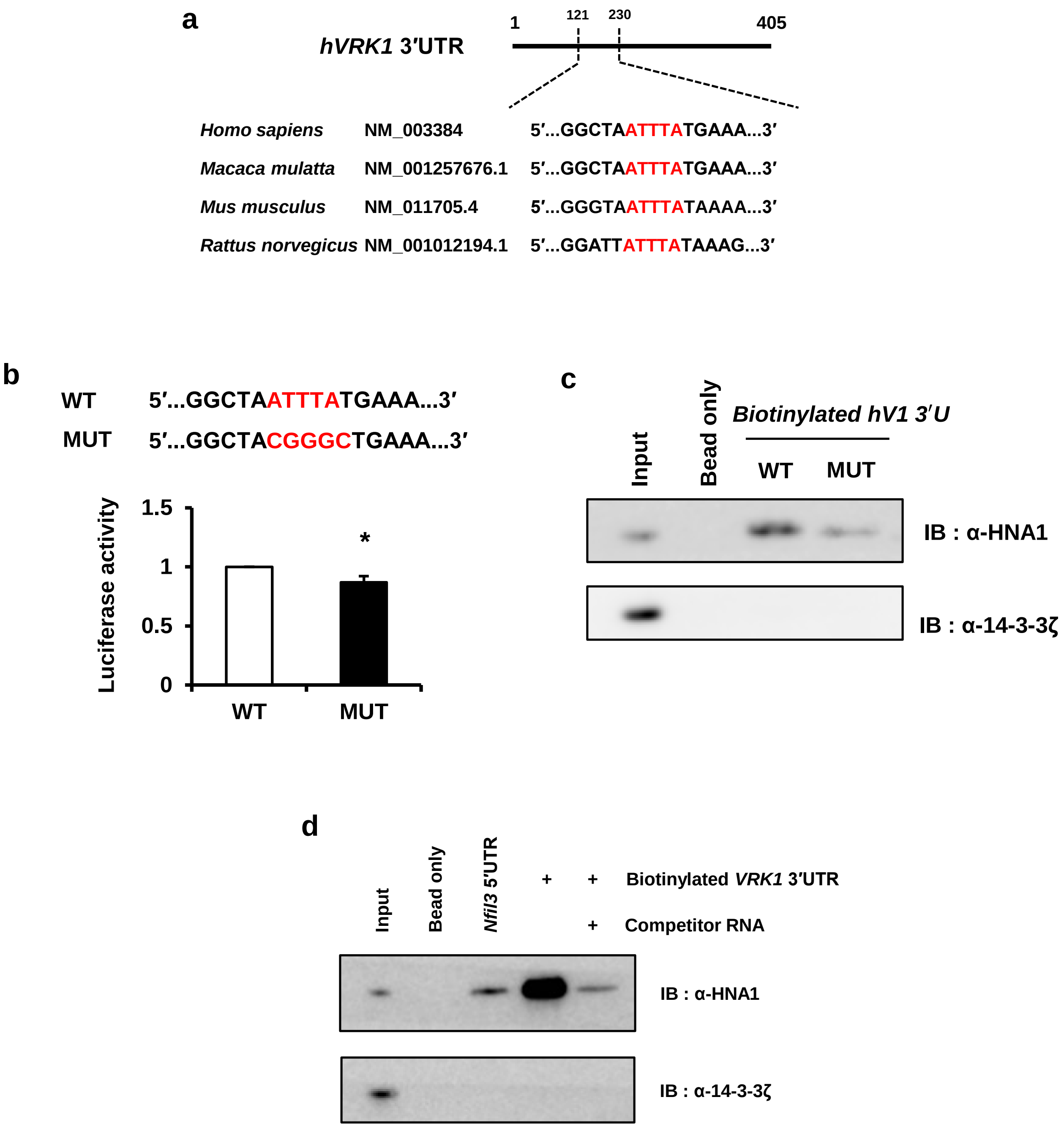

a

**Putative HNRNP A1 interacting proteins** (BioGRID - Database of Protein, Chemical, and Genetic Interaction)
HNRNP R, H1, A2B1, H3, L, I, DL, K, M, Q, UL2, U, C, D, AB, F, H2, A1L2, A3, A0, A1, UL1, A1P70, CL2
ELAVL1, RBMX, EWSR1, RBMS1, RBM14, UPF1, RBM3, NCL, RBM6, RBM12B, FUS, RBM7, RBM12, RBM26, RBM39, YBX3, FUS, STAU1
MOV10, DHX9, DDX17, DDX5, DHX30, DDX1, DHX36, DDX28, DHX15, DDX23, DDX21, DDX6, DDX3X
PABPC1, NCBP1, PAIP2B, PAIP2, PABPC4, PCBP2
EEF1A1, EIF4EBP1, EIF4A2, EIF3H, EIF2B3, EIF2B1, EEF2, EEF1G
RPS3A, RPS27A, RPS15, RPS3, RPS15A, RPS21, RPS5, RPLP2, RPSA, RPS6, RPAP1, RPL38, RPL27A, RPS7

Figure S4. Ryu *et al.*

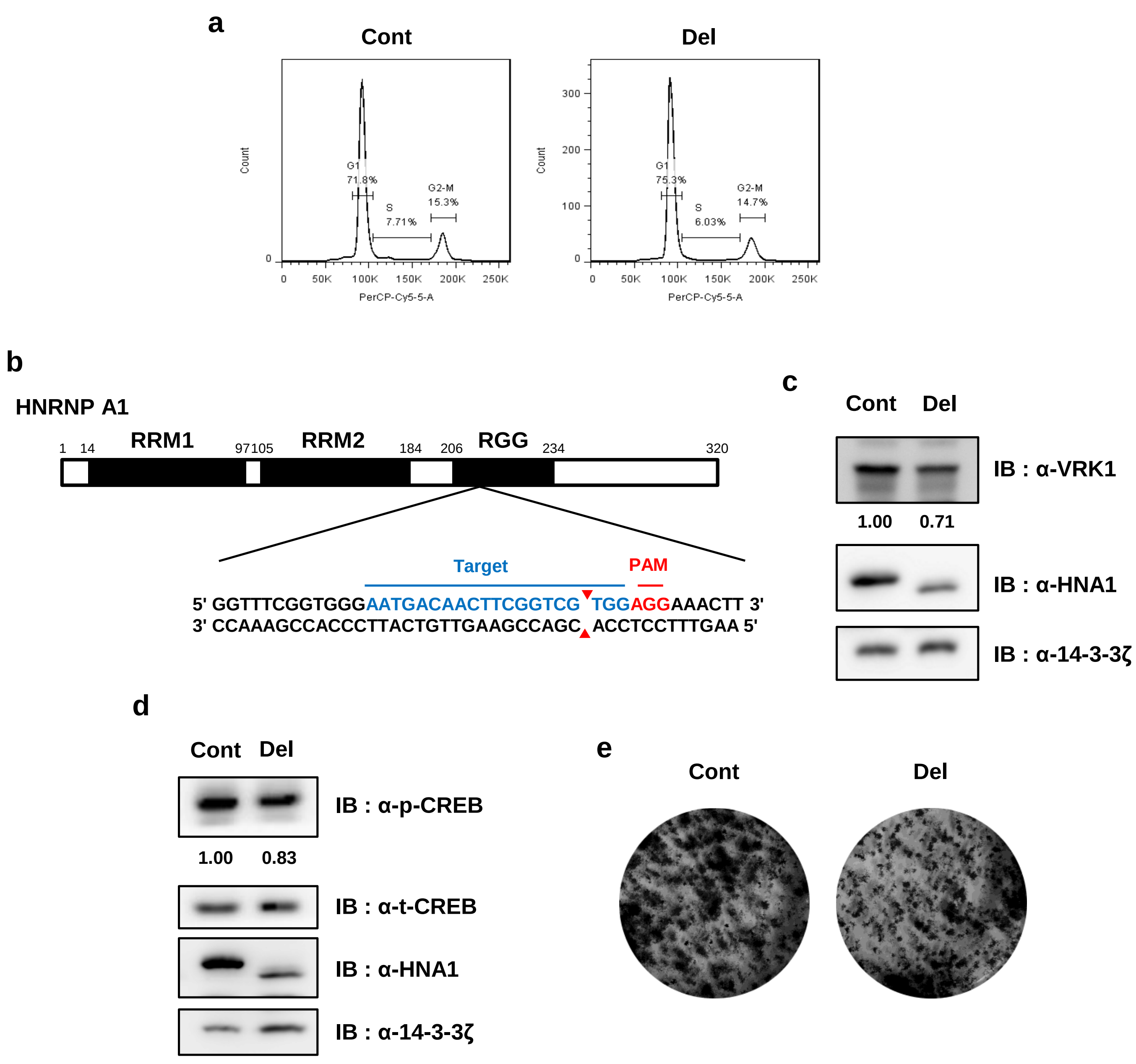

Figure S5. Ryu *et al.*

a

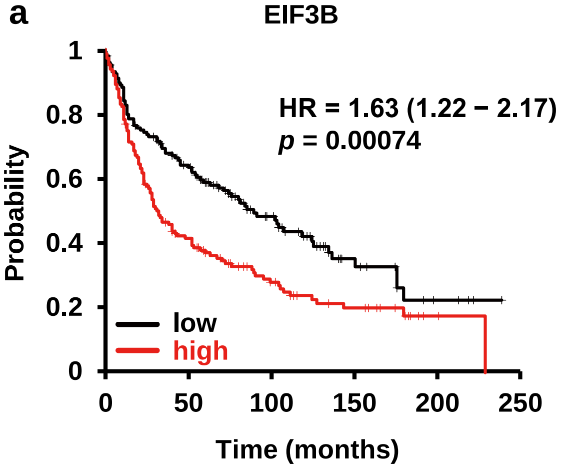

Supplementary Figure S1. HNRNP A1 regulates the *VRK1* mRNA translation in H1299 cells. (a) Western blot analysis of the levels of VRK1, HNRNP A1 and 14-3-3ζ in H1299 cells 48 h after siRNA transfection. (b) *VRK1* mRNA levels in H1299 cells transfected with either si_control (si_c) or HNRNP A1-targeting siRNA (si_HNA1) were quantified by qRT-PCR analysis (unpaired two-tailed Student's *t*-test, *n*=3; n.s., not significant). (c) Levels of reporter activities are measured by analysis of *VRK1* 3′UTR (V1 3′U) luciferase reporter. Luciferase activity is shown as the ratio of hRluc to hluc+, and the luciferase activity from mock vector was set as 1. Bars represent means±s.e.m. (unpaired two-tailed Student's *t*-test, *n*=5; $^{*}P<0.05$). (d) HNRNP A1 stably expressing cells were generated. HNRNP A1 stably expressing H1299 cell lysates were subjected to immunoblotting with the indicated antibodies. The arrowhead indicates NTAP-HNRNP A1, while the asterisk indicates endogenous HNRNP A1. (e) Western blot analysis of the levels of VRK1, HNRNP A1 and 14-3-3ζ in H1299 stable cells is performed. (f) *VRK1* mRNA levels in HNRNP A1 stably expressing cell line were quantified by qRT-PCR analysis (unpaired two-tailed Student's *t*-test, *n*=3; n.s., not significant). (g) Levels of reporter activities are measured by analysis of *VRK1* 3′UTR (V1 3′U) luciferase reporter. Luciferase activity is shown as the ratio of hRluc to hluc+, and the luciferase activity from mock vector was set as 1. Bars represent means±s.e.m. (unpaired two-tailed Student's *t*-test, *n*=5; $^{*}P<0.05$). (h) *VRK1* mRNA and *TBP* mRNA levels after 2, 4 and 6 h of actinomycin D (Act.D) treatment are shown as percentages on the y-axis (*n*=2~3).

Supplementary Figure S2. The conserved regions in the *VRK1* 3′UTR is a target of HNRNP A1. (a) The HNRNP A1 binding site in the 3′UTR region of *VRK1* was highly conserved among several species. Red letters indicate the conserved sequence of *VRK1* and the target of HNRNP A1. (b) Levels of reporter activities are measured by analysis of *VRK1* WT 3′UTR (WT) and *VRK1* mutated 3′UTR (MUT) luciferase reporters. Luciferase activity from WT vector was set as 1. Bars represent means±s.e.m. (unpaired two-tailed Student's *t*-test, *n*=5; $^{*}P<0.05$). (c-d) The *in vitro* transcribed *VRK1 WT* 3′UTR and *mutated* 3′UTR constructs were labelled with biotin-UTP and were incubated with HEK293A cell extract. Biotin-UTP labelled RNAs were pulled down with streptavidin bead. Streptavidin-affinity purified samples were separated by SDS–PAGE and were subjected to immunoblotting with anti-HNRNP A1. Abundance of HNRNP A1 on *VRK WT* 3'UTR was decreased in the reaction with biotin-labelled *mutated* 3′UTR mRNA. 14-3-3ζ was used as negative control.

Supplementary Figure S3. *In silico* prediction of HNRNP A1 interacting proteins. (a) List of physical protein-protein interaction as a subset of the BioGRID database.

Supplementary Figure S4. Deletion of *cis*-acting element or HNRNP A1 RGG domain leads to disruptions of cell growth. (a) Cell cycle analysis of *cis*-acting region deleted cells. The number of *cis*-acting region deleted cells at G1, S and G2/M phase was quantified. (b) HNRNP A1 RGG domain mutant cells were generated using CRISPR/Cas9 system. (c and d) Immunoblots for VRK1 and p-CREB in HNRNP A1 RGG domain deleted cell lines. 14-3-3ζ was used as a loading control. Numbers indicate densitometric values determined by VRK1/14-3-3ζ or p-CREB/t-CREB ratios. The average value of densitometry in Control cells was set to 1. (e) Colony formation assays for the designated cell lines were performed.

Supplementary Figure S5. EIF3B is negatively correlated to survival. (a) The Kaplan–Meier curve showing overall survival of patients with lung cancers bearing high or low *EIF3B* expression is graphed.
